# Human Visceral Leishmaniasis in Kermanshah Province, Western Iran, During 2011-2012

**Published:** 2012

**Authors:** Y Hamzavi, B Hamzeh, M Mohebali, B Akhoundi, Kh Ajhang, N Khademi, K Ghadiri, H Bashiri, M Pajhouhan

**Affiliations:** 1 Department of Medical Parasitology and Mycology, School of Medicine, Kermanshah University of Medical Sciences, Kermanshah, Iran; 2 Department of Statistic & Epidemiology, School of Public Health, Kermanshah University of Medical Sciences, Kermanshah, Iran; 3 Department of Medical Parasitology and Mycology, School of Public Health, Tehran University of Medical Sciences, Tehran, Iran; 4 Center for Research of Endemic Parasites of Iran (CREPI), Tehran University of Medical Sciences, Tehran, Iran; 5 Province Health Center, Kermanshah University of Medical Sciences, Kermanshah, Iran; 6 Department of Pediatrics, School of Medicine, Kermanshah University of Medical Sciences, Kermanshah, Iran; 7 Department of Internal Medicine, School of Medicine, Kermanshah University of Medical Sciences , Kermanshah, Iran; 8 Laboratory Sciences, Kermanshah University of Medical Sciences, Kermanshah, Iran

**Keywords:** Visceral leishmaniasis, Kala-azar, Seroprevalence, Seroepidemiology, Iran

## Abstract

**Background:**

Visceral leishmaniasis (VL) or kala-azar is a parasitic disease caused by the species of *Leishmania donovani complex*. It is endemic in some parts of provinces of Iran. According to the reported cases of VL in Kermanshah Province in recent years, this study was conducted to determine the seroprevalence of VL in high risk villages of the province.

**Methods:**

Totally, 1622 serum samples obtained from children under 15 years old and 178 from adults in 22 villages of studied areas. Serum samples were examined by direct agglutination test (DAT) for the detection of anti-*Leishmania* antibodies. Data were analyzed using SPSS software ver.11.5.

**Results:**

Only 6 serum samples (0.33%) showed anti-*Leishmania* antibodies against *L.infantum* at titers ≥ 1/3200. Four of the seropositive cases had a history of kala-azar and Leishman bodies were seen in their bone marrows. The highest (0.5%) and lowest (0.29%) seroprevalence was seen in the age groups of 5-9 and 10-14 years old, respectively. None of the adults were seropositive. There were not any significant differences between the rate of seropositivity in males (0.36%) and females (0.31%). 66.7% of seropositive individuals showed clinical manifestations. The most important symptoms in Kala-azar patients were fever, hepato-spleenomegally and anemia.

**Conclusion:**

Kala-azar is occurred sporadically in Kermanshah Province. But presence of significant number of positive sera confirms the necessity for attention of people and clinicians to kala-azar.

## Introduction

Kala-azar / visceral leishmaniasis (VL) is an important protozoan disease usually caused by *Leishmania donovani complex*. VL is expanded in the Old and New Worlds and transmitted by sand flies. It is estimated that the annual occurrence of human VL is 500,000 worldwide (1). Mediterranean VL is endemic in some parts of Iran, including Ardabil (Meshkin shahr and Moghan), eastern Azerbaijan (Kalibar and Ahar), Fars (Firouz abad, Jahrom, Noor abad and Darab), Bushehr (Dashti and Dashtestan) and Qom (Khaljestan) districts. In other provinces of Iran the disease has been reported in sporadic form (2–7). Altogether, more than 3000 cases of VL had been diagnosed in 31 Iranian provinces up to 2010. About 40% of VL cases are reported from northwestern Iran. The average annual number of the diagnosed cases of VL in Iran during last decade was 0.449 cases/100,000 inhabitants. The highest incidence rate of VL was 57 cases/100,000 inhabitants from Ardabil Province, northwestern Iran (8).

Canine are the major reservoir hosts of Mediterranean VL. Canine *L. infantum* infections were determined to be 14.2%, 17.4% and 21.6% in different parts of Iran (9–12). Both symptomatic and asymptomatic infected dogs are the most important source of infection for human (1, 5, 13). Sand flies are the vectors of parasite and natural leptomonad infections were observed in four species of phlebotomus sand flies in some parts of Iran (14–16).

Recently Keyghobadi et al. reported 8 cases of kala-azar disease in some areas of Kermanshah Province from 2005-2008. These 8 patients were from areas such as Javanroud, Paveh, Sarpol-zahab, Ravansar and Dalahoo (17). From 1990-94, five children and from 2004-2009 nine children, have been diagnosed as kala-azar disease in Kermanshah hospitals. *L. infantum* has been reported as the causative agent of disease using PCR technique (18).

An appropriate serological test developed for field use is the direct agglutination test (DAT) as a quantitative test. This test has been extensively validated in most endemic areas (19–22). DAT, was developed and described for serodiagnosis and sero-epidemiological studies of VL (19, 20). DAT was also modified and used as a simple, reliable, cost-effective and suitable tool for the diagnosis and sero-epidemiological surveys of VL in human and canine reservoir hosts of the disease in Iran (9, 23, 24).

Because there is not any information about seroprevalence of VL in Kermanshah Province, western Iran this study aimed to determine the seroprevalence of VL using DAT in high risk villages of the province.

## Materials and Methods

### Study area

Kermanshah Province is situated in the western slope of the Zagros range of mountains in the west of Iran. The province with an area of 24,361 square kilometers contains almost 1.6% of the total land of the country, and with population of 1,938,060 has about 2.5% of total population of the country. About 61.75% of the population is in urban areas, 37.7% in rural areas and less than1% are nomadic (25).

**Fig. 1:**
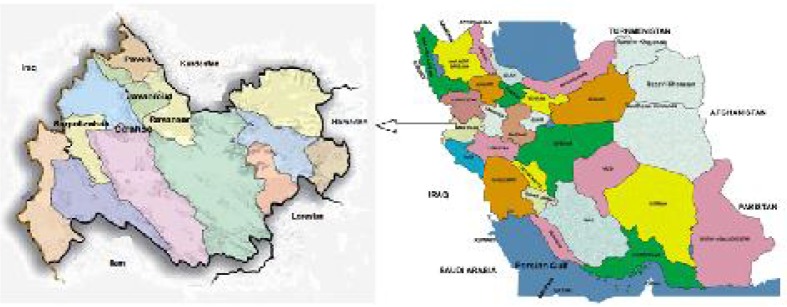
Situation of Kermanshah in Iran and location of study areas in Kermanshah Province, west of Iran

The investigation was carried out over a period of 18 months from September 2011 to April 2012 in some of high risk villages of five districts of the province such as Javanroud, Paveh, Sarpol-zahab, Ravansar and Dalahoo. We selected 22 villages for study which the disease have been reported from them in the last years (Fig. 1).

### Blood collection

A questionnaire was filled out for each case including various factors such as age, sex, locality etc. An informed consent was taken from all of the adult subjects or parents of children. Questionnaires were completed by trained health workers in the health houses and trained technicians in the rural health centers (RHCs) and district health centers (DHCs). Blood samples were collected in heparinized capillary tubes from 1622 children under 15 years old and 178 adults. The collected blood samples were centrifuged at 800 g for 5-10 min and the sera/plasma were separated. Samples transferred to the parasitology research laboratory in faculty of medicine of Kermanshah University of Medical Sciences in a cold box and stored in -70 °C.

### Preparation of DAT antigen and performance of DAT

DAT antigen was prepared in the Protozoology unit of the School of Public Health, Tehran University of Medical Sciences. Antigen prepared by mass production of promastigotes of Iranian strain of *L. infantum* [MCAN/IR/07/Moheb-gh. (GenBank accession no. *FJ555210*)] in RPMI-1640 medium (Biosera, South America) plus 10% fetal calf serum (Biosera, South America), following tripsinization of the parasites, staining with Coomassie brilliant blue R-250 (Sigma, USA) and fixing with formaldehyde 1.2% (9, 19, 20, 23).

Direct agglutination test (DAT) was used for seroprevalence of VL. Serum/plasma samples were diluted 1:10 to 1:3200 in a V-shaped micro-titer plate into a dilution fluid containing 0.9% saline and 0.78% 2-mercaptoethanol. One equal volume (50 ml) of antigen suspension was added to each well. The results were read after 18-24 h incubation in a wet room at room temperature. The highest dilution at which agglutination was still visible in comparison with positive and negative controls titer was defined as the titer of sample. Compact blue dots were scored as negative and large diffuse blue mats as positive. Titers of ≥ 1:3200 were considered as seropositive (24, 26). The sero-positive cases were referred to pediatrician for physical examination and received appropriate treatment if necessary. Data were analyzed using SPSS software ver.11.5.

## Results

Studied villages for detection of seroprevalence of human visceral *Leishmania* infection in Kermanshah Province is shown in Table (1). Totally 1800 serum samples collected from 22 villages in five districts.

**Table 1:** Study villages for detection of seroprevalence of human visceral *Leishmania* infection in Kermanshah Province, 2011-2012

District	Villages	No. of sera
Dalahoo	Yaran-olia,yaran sofla,Ghelgheleh,Jhalkeh	190
Paveh & Javanroud	Kalash luolem,Kalash lameh,Kalash ghoveh,Marzan,galileh,Baneh werah,Sar Koran,Chou zari,Ordougah,Sar yas	600
Ravansar	Masour aghaei,Gheshlagh,Tazeh abad	640
Sarpol-zahab	Ramaki sofla,Ramaki olia,Jawanmiri,Bani howan,Hasan solaiman	370
**Total**		1800

Age and gender distribution of samples can be seen in Table (2). About 53.6% of samples were collected from males and 46.4% from females. The most and the least samples were collected from age groups of 10-14 years old (38.6%) and adults ≥ 15 years old (9.9%) respectively.

Frequency of anti-*Leishmania* antibody titers with DAT according to the age groups can be seen in Table (3). Only six cases had titers ≥ 1:3200. The highest percentage of seropositivity was seen in age groups of 5-9 (0.5%) and 0-4 (0.31%) years old respectively.

**Table 2:** Distribution of studied population for detection of seroprevalence of human visceral *Leishmania infantum* infection by gender and age groups in Kermanshah Province, 2011-2012

Age groups (yr)	Males		Females		Total	
	
	No. of sera	%	No. of sera	%	No. of sera	%
0-4	178	55.3	144	44.7	322	17.9
5-9	314	51.9	291	48.1	605	33.6
10-14	346	49.8	349	50.2	695	38.6
≥15	127	71.3	51	28.7	178	9.9
Total	965	53.6	835	46.4	1800	100

**Table 3:** Seroprevalence of human visceral *Leishmania* infection by direct agglutination test (DAT ≥ 1:3200) with anti-*Leishmania infantum* antibodies by age groups in Kermanshah Province, 2011-2012

Age groups (yr)		Antibody Titer						Total	
		
		1:800		1:1600		≥1:3200			
		
	No. of sera	No. of sera	%	No. of sera	%	No. of sera	%	No. of sera	%
0-4	322	2	0.62	1	0.31	1	0.31	4	1.24
5-9	605	2	0.33	1	0.16	3	0.50	6	0.99
10-14	695	3	0.43	2	0.29	2	0.29	7	1
≥15	178	2	1.12	0	0	0	0	2	1.12
Total	1800	9	0.50	4	0.22	6	0.33	19	1.05

Table (4) shows titers of anti-*Leishmania infantum* antibodies in six seropositive cases according to the age, Gender, city and village of residence in the province. The most cases of seropositive cases were seen in Sarpol-zahab, Paveh and Javanrood, respectively. Overall as shown in Table 5, about 0.31% of cases with anti-*Leishmania* antibody titers ≥1:3200 were in males and 0.36% in females.

**Table 4:** Anti-*Leishmania infantum* antibody titers of six seropositive cases of visceral *Leishmania* infection by direct agglutination test with respect to their age, gender and locality in Kermanshah Province, 2011-2012

Case No.	Age (yr)	Gender	District	Village	Antibody Titer
1	5	Female	Paveh	Baneh werah	1:3200
2	12	Female	Sarpol-zahab	Bani howan	1:3200
3	8	Female	Sarpol-zahab	Jawanmiri	1:6400
4	6	Male	Javanrood	Kalash luolem	1:12800
5	3	Male	Sarpol-zahab	Jawanmiri	1:3200
6	10	Male	Paveh	Baneh werah	1:3200

**Table 5:** Seroprevalence of human visceral *Leishmania* infection by direct agglutination test (DAT ≥ 1:3200) with anti-*Leishmania infantum* antibodies by gender in Kermanshah Province, 2011-2012

Gender	No. of sera	Antibody Titer						Total	
		
		1:800		1:1600		≥1:3200			
		
		No. of sera	%	No. of sera	%	No. of sera	%	No. of sera	%
**Male**	965	5	0.51	2	0.20	3	0.31	10	1.03
**Female**	835	4	0.49	2	0.24	3	0.36	9	1.07
**Total**	1800	9	34.62	4	15.38	6	50	19	1.05

66.7% of seropositive individuals showed clinical manifestations. The most important symptoms in Kala-azar patients were fever, hepato-spleenomegally and anemia. Four of six cases with titers of ≥1:3200 had a history of kala-azar disease in recent years and Leishman bodies had been seen in their bone marrows. But two others (33.3%) did not have any history of disease. Review of hospital records of Kala-azar patients in the province in the past years, revealed that the main symptoms were fever (89%), hepato-splenomegaly (79%) and anemia (75.5%).

## Discussion

Despite the diversity of climate and ecological conditions and the presence of numerous cases of cutaneous leishmaniasis in Kermanshah Province (27–29), there was not any information about status of visceral leishmaniasis in this province. This is the first study which detects the seroprevalence of VL in Kermanshah Province and gives us some important information about this disease in the province.

Analysis of data showed that about 0.33% of population were seropositive and had anti-*Leishmania* antibodies at titers of ≥1:3200. During 2002-2005 the seroprevalence of VL (DAT ≥ 1:3200) in some parts of the country were as follows: Ardabil Province [Germi (2.8%), Meshkinshahr (6.3%), Ardabil, Pars-Abad, and Khalkhal(5.1%)], Chahar Mahal & Bakhtiari province[Koohrang (2.3%)], Fars Province [Mamasani (1.9%)], Lorestan Province [Poshtkuh (1.3%)], Kohgiloyeh & Bouir ahmad Province [Yasuj (1.5%)], Khorasan Province [ Bojnurd and Shirvan, (46/0%)] (23). Hamzavi et al. found 3.4% of seropositivity in Dashti and Dashtestan Districts of Bushehr Province (5). Fakhar et al. found 1.7% of seropositivity in 8 villages of Ghahan from Qom Province that three of seven seropositive cases had a previous history of VL (7). Results showed that the rate of seropositivity in Kermanshah is fewer than these areas.

The most cases of seropositive cases were seen in Sarpol-zahab (50%). The rate of seropositivity in Paveh and Javanrood was 33.3% and 1.7% respectively. It seems that the importance of VL in these areas is more than other parts of the province. Some cases of VL in the villages of Sarpol-zahab and Javanrood in the past years have been reported (18).

The most seropositivity rate (0.5%) was seen in the age group of 5-9 years old. Presence of two cases with previous history of VL in this age group may be a reason for elevation of seropositivity in this age group. 66.7% and 83.3% of seropositive cases were in the age groups of 0-9 and 0-12 years old respectively. Prior studies in Iran had shown a seroprevalence rate of about 50% in the age group of 1-2 years old and 96% of sero-positive cases were in children up to 8 years of age (2). In endemic areas of Iran 98% of VL were observed in the children under ten years old and most of them were in rural communities and nomadic tribes (9).

0.31% and 0.36% of seropositive cases was seen in males and females respectively. Therefore, it does not seem that VL affects females more than males, at least in Kermanshah Province. In earlier study in Bushehr Province we found 3.8% and 3.0% of seropositivity in males and females respectively, and no statistically significant difference was observed between them (5). In some rural areas the rate of active kala-azar cases in males may be higher than females but this difference was not statistically significant (22).

In this study fever, hepato-splenomegaly and anemia were predominant clinical features. The most predominant clinical features of 142 diagnosed kala-azar cases found in the report of Mohebali et al. were fever, weakness, paleness, and hepato-splenomegaly. These signs and symptoms are the same as those found in other clinical studies (22). Kala-azar in Iran has been reported from asymptomatic to severe and fatal cases. In some parts of Iran asymptomatic forms is much more than symptomatic disease (5, 6, 30), and may be more than 32% of seropositive individuals require no treatment (31). Four of the six seropositive cases (66.6%) had a history of kala-azar. The other two cases (33.4%) did not have any signs or symptoms and can be considered as asymptomatic form.

In Iran, constantly new foci of VL have been identified in the past years. Emerging of VL in different parts of the country needs to rapid diagnosis and treatment of disease, which can reduce the incidence of severe illness and death. Due to the emergence of VL in some parts of the province, it should be necessary that clinicians have more attention to VL in differential diagnosis of diseases. On the other hand it is important to increase the awareness of people, especially in high risk parts of the province to refer to appropriate health and medical centers whenever persistent fever, anemia and a large abdomen developed in their children.

## Conclusion

Kala-azar in Kermanshah Province is a sporadic disease and the rate of seropositivity in the province is fewer than other examined parts of the country. But importance of VL in some parts located in the north-west of the province is more than other parts of the province. Study on the epidemiology, vectors and probable sources of disease are recommended.
